# Trends in all-cause mortality during the scale-up of an antiretroviral therapy programme: a cross-sectional study in Lusaka, Zambia

**DOI:** 10.2471/BLT.13.134239

**Published:** 2014-06-26

**Authors:** Sujit D Rathod, Benjamin H Chi, Thankian Kusanthan, Batista Chilopa, Jens Levy, Izukanji Sikazwe, Peter Mwaba, Jeffrey SA Stringer

**Affiliations:** a Department of Population Health, London School of Hygiene and Tropical Medicine, Keppel Street, London WC1E 7HT, England.; b Centre for Infectious Disease Research in Zambia, Lusaka, Zambia.; c University of Zambia, Lusaka, Zambia.; d Zambian Central Statistical Office, Lusaka, Zambia.; e Zambian Ministry of Health, Lusaka, Zambia.

## Abstract

**Objective:**

To follow the trends in all-cause mortality in Lusaka, Zambia, during the scale-up of a national programme of antiretroviral therapy (ART).

**Methods:**

Between November 2004 and September 2011, we conducted 12 survey rounds as part of a cross-sectional study in Lusaka, with independent sampling in each round. In each survey, we asked the heads of 3600 households to state the number of deaths in their households in the previous 12 months and the number of orphans aged less than 16 years in their households and investigated the heads’ knowledge, attitudes and practices related to human immunodeficiency virus (HIV).

**Findings:**

The number of deaths we recorded – per 100 person–years – in each survey ranged from 0.92 (95% confidence interval, CI: 0.78–1.09) in September 2011, to 1.94 (95% CI: 1.60–2.35) in March 2007. We found that mortality decreased only modestly each year (mortality rate ratio: 0.98; 95% CI: 0.95–1.00; *P* = 0.093). The proportion of households with orphans under the age of 16 years decreased from 17% in 2004 to 7% in 2011. The proportions of respondents who had ever been tested for HIV, had a comprehensive knowledge of HIV, knew where to obtain free ART and reported that a non-pregnant household member was receiving ART gradually increased.

**Conclusion:**

The expansion of ART services in Lusaka was not associated with a reduction in all-cause mortality. Coverage, patient adherence and retention may all have to be increased if ART is to have a robust and lasting impact at population level in Lusaka.

## Résumé

**Objectif:**

Suivre les tendances dans la mortalité toutes causes confondues à Lusaka, en Zambie, au cours du développement du programme national de traitement antirétroviral (TAR).

**Méthodes:**

Entre novembre 2004 et septembre 2011, nous avons mené 12 cycles d'études dans le cadre d'une étude transversale à Lusaka, avec un échantillonnage indépendant à chaque cycle. Dans chaque étude, nous avons demandé aux chefs de famille de 3600 ménages de déclarer le nombre de décès qui avaient eu lieu dans leur famille au cours des 12 derniers mois et le nombre d'orphelins âgés de moins de 16 ans dans leur famille, et nous avons examiné les connaissances, les attitudes et les pratiques liées au virus de l'immunodéficience humaine (VIH) des chefs de famille.

**Résultats:**

Le nombre de décès que nous avons enregistrés – pour 100 personnes par an – dans chaque étude est compris entre 0,92 (intervalle de confiance de 95%, IC: 0,78–1,09) en septembre 2011 et 1,94 (IC de 95%: 1,60–2,35) en mars 2007. Nous avons constaté que la mortalité ne diminuait que modestement chaque année (rapport du taux de mortalité: 0,98; IC de 95%: 0,95–1,00; *P* = 0,093). Le pourcentage des ménages avec des orphelins de moins de 16 ans a diminué de 17% en 2004 à 7% en 2011. Le pourcentage des répondants qui avaient déjà été dépistés pour le VIH, qui avaient une connaissance approfondie du VIH, qui savaient où obtenir un TAR gratuit et qui avaient déclaré qu'une femme non enceinte du ménage bénéficiait d'un TAR, a progressivement augmenté.

**Conclusion:**

Le développement des services de TAR à Lusaka n'était pas associé à une réduction de la mortalité toutes causes confondues. La couverture, l'adhésion des patients et la rétention pourraient être toutes améliorées si le TAR devait avoir un impact fort et durable au niveau de la population à Lusaka.

## Resumen

**Objetivo:**

Seguir las tendencias de la mortalidad general en Lusaka (Zambia) durante la ampliación de un programa nacional de una terapia antirretroviral (TARV).

**Métodos:**

Como parte de un estudio transversal, realizamos 12 encuestas con un muestreo independiente en cada una de ellas entre noviembre de 2004 y septiembre de 2011 en Lusaka. En cada encuesta, se solicitó a los cabezas de familia de 3600 hogares que indicaran el número de muertes en los últimos 12 meses y el número de huérfanos menores de 16 años en sus hogares y se investigaron los conocimientos, actitudes y prácticas de los cabezas de familia en relación con virus de la inmunodeficiencia humana (VIH).

**Resultados:**

El número de muertes registradas – por 100 personas al año – en cada encuesta varió de 0,92 (intervalo de confianza del 95%, IC: 0,78–1,09) en septiembre de 2011 a 1,94 (IC del 95%: 1,60–2,35) en marzo de 2007. Descubrimos que la mortalidad solo se redujo ligeramente cada año (razón del índice de mortalidad: 0,98; IC del 95%: 0,95–1,00; P = 0,093). La proporción de hogares con huérfanos menores de 16 años disminuyó del 17% en 2004 al 7% en 2011. La proporción de los encuestados que nunca se había sometido a las pruebas del VIH, que tenía un conocimiento amplio sobre el VIH, que sabía dónde obtener tratamiento antirretroviral gratuito y que informó de que un miembro no embarazado del hogar recibía TARV aumentó gradualmente.

**Conclusión:**

La expansión de los servicios de TARV en Lusaka no estuvo asociada a una reducción en la mortalidad general. Si se pretende que la TARV tenga un efecto importante y duradero en el nivel de población de Lusaka, es posible que sea necesario reforzar la cobertura, la adherencia y la retención de los pacientes.

## ملخص

**الغرض:**

تتبع الاتجاهات في الوفيات الناجمة عن جميع الأسباب في لوساكا، بزامبيا أثناء زيادة حجم البرنامج الوطني للعلاج بمضادات الفيروسات القهقرية (ART).

**الطريقة:**

قمنا بين تشرين الثاني/نوفمبر 2004 وأيلول/سبتمبر 2011 بإجراء 12 جولة مسح كجزء من دراسة متعددة القطاعات في لوساكا، حيث تم أخذ عينات مستقلة في كل جولة. وطلبنا في كل ميح من أرباب 3600 أسرة بيان عدد الوفيات في أسرهم خلال الاثني عشر شهراً الماضية وعدد اليتامى الأقل من 16 سنة في أسرهم وتحرينا معرفة أرباب الأسر واتجاهاتهم وممارساتهم ذات الصلة بفيروس العوز المناعي البشري (HIV).

**النتائج:**

تراوح عدد الوفيات التي قمنا بتسجيلها - لكل 100 شخص في السنة - في كل مسح من 0.92 (فاصل الثقة 95 %، فاصل الثقة: 0.78 - 1.09) في أيلول/سبتمبر 2011 إلى 1.94 (فاصل الثقة 95 %، فاصل الثقة: 1.60 - 2.35) في آذار/مارس 2007. وتوصلنا إلى وجود انخفاض متواضع فقط في معدل الوفيات كل سنة (نسبة معدل الوفيات: 0.98؛ فاصل الثقة 95 %، فاصل الثقة: 0.95 - 1.00، الاحتمال = 0.093). وانخفضت نسبة الأسر التي تضم أيتاماً دون سن 16 سنة من 17 % في عام 2004 إلى 7 % في عام 2011. وازدادت تدريجياً نسب المجيبين الذين خضعوا لاختبار فيروس العوز المناعي البشري وكانت لديهم معرفة شاملة بفيروس العوز المناعي البشري ومعرفة بأماكن الحصول على العلاج المجاني بمضادات الفيروسات القهقرية وقاموا بالإبلاغ عن تلقي أحد أفراد الأسرة من النساء غير الحوامل العلاج بمضادات الفيروسات القهقرية.

**الاستنتاج:**

لم يرتبط توسع خدمات العلاج بمضادات الفيروسات القهقرية في لوساكا بانخفاض في الوفيات الناجمة عن جميع الأسباب. وقد يتعين زيادة التغطية وامتثال المرضى والإبقاء عليهم إذا كانت هناك رغبة في تحقيق أثر قوي ومستمر للعلاج بمضادات الفيروسات القهقرية على صعيد السكان في لوساكا.

## 摘要

**目的:**

跟踪在扩大抗逆转录病毒治疗国家计划（ART）中赞比亚卢萨卡的全因死亡率的趋势。

**方法:**

在2004年11月至2011年9月之间，作为卢萨卡横断面研究的组成部分，我们进行了12轮的调查，每一轮均独立抽样。我们在每次调查中询问了3600户家庭负责人其家庭在最近12个月中的死亡人数、家庭中16岁以下孤儿人数并调查负责人艾滋病（HIV）的相关知识、态度和实践。

**结果:**

我们在每个调查中记录的死亡数（每100人年）在2011年 9月的0.92（95%置信区间，CI：0.78-1.09）到2007年3月的1.94（95% CI：1.60-2.35）的范围。我们发现每年死亡率仅有略微降低（死亡率比：0.98；95% CI：0.95-1.00；*P* = 0.093）。有16岁以下孤儿的家庭比例从2004年的17%下降到2011年的7%。受访者曾经进行过艾滋病毒检测、具有艾滋病的综合知识、知道在何处接受免费ART治疗并报告非妊娠家庭成员正在接受ART治疗的比例逐渐增加。

**结论:**

卢萨卡ART服务扩张与全因死亡率降低无关联。如果要使ART治疗对卢萨卡人口水平保持强大持久的影响，可能必须提高覆盖率、病人依循性和保持情况。

## Резюме

**Цель:**

Проследить тенденции общей смертности в Лусаке, Замбия, при расширении масштабов национальной программы антиретровирусной терапии (АРТ).

**Методы:**

В период с ноября 2004 года по сентябрь 2011 года были проведены 12 раундов опросов в рамках перекрестного исследования в Лусаке с независимыми выборками в каждом раунде. В ходе каждого опроса от глав 3600 домохозяйств требовалось сообщить число смертей в течение предыдущих 12 месяцев и число сирот в возрасте до 16 лет в своих домохозяйствах, а также было проведено исследование знаний, отношения и практик глав домохозяйств, связанных с вирусом иммунодефицита человека (ВИЧ).

**Результаты:**

Число зарегистрированных смертей на 100 человеко-лет в каждом опросе варьировалось от 0,92 (95%-й доверительный интервал, ДИ: 0,78–1,09) в сентябре 2011 года до 1,94 (95% ДИ: 1,60–2,35) в марте 2007 года. Было обнаружено, что смертность снижалась каждый год лишь незначительно (коэффициент смертности: 0,98; 95% ДИ: 0,95–1,00; *P* = 0,093). Доля домохозяйств с сиротами в возрасте до 16 лет снизилась с 17% в 2004 году до 7% в 2011 году. Постепенно увеличивалась доля респондентов, которые когда-либо проходили тест на ВИЧ, обладали всесторонними знаниями о ВИЧ, знали, где получить бесплатную АРТ, и сообщили, что небеременные члены домохозяйств получали АРТ.

**Вывод:**

Расширение услуг по АРТ в Лусаке не было связано со снижением общей смертности. Такие показатели, как охват, соблюдение пациентами указаний и уровень удержания пациентов, могут увеличиться, если АРТ окажет устойчивое и продолжительное воздействие на уровень популяции в Лусаке.

## Introduction

The individual health benefits associated with antiretroviral therapy (ART) are well established^1^^,^^2^ and treatment of human immunodeficiency virus (HIV) infections is now recommended worldwide.^3^ Over the past decade, governments and donors have invested heavily in control and treatment of HIV and acquired immunodeficiency syndrome (AIDS). ART programmes have been expanded under the assumption that – given sufficient coverage – ART can reduce HIV-related mortality and reverse the rise in all-cause mortality that has been frequently associated with increasing prevalence of HIV infection.^4^^,^^5^

In several studies in sub-Saharan Africa, reductions in population-level mortality have followed the introduction of ART services.^6^^–^^12^ While encouraging, these studies only followed the short-term impact of the initiation of an ART programme and none was conducted solely in an urban setting where HIV prevalence was high.

The prevalence of HIV among adults living in Zambia was estimated to be 12.5% in 2011.^13^^,^^14^ Since 2004, the Zambian Ministry of Health has being scaling up a programme for HIV care and treatment across the country’s nine provinces. By 2011, 80% of the Zambians who were eligible for ART – more than 400 000 people – were receiving treatment.^15^ In the capital city of Lusaka, which has a population of about 1.7 million,^16^ HIV treatment in the public sector is currently available at 19 primary health centres and a tertiary care hospital. Between November 2004 and June 2011 – as public sector ART services were scaled up – we implemented a population-based, repeated cross-sectional study in Lusaka district. Our primary hypothesis was that, as the expanding public ART programme covered an ever larger proportion of the population, we would observe a reduction in all-cause mortality. We also sought to describe the trends in HIV-related knowledge, attitudes and practices and the trends in the presence of orphans under the age of 16 in the city’s households.

## Methods

The implementation of our study, which involved 12 rounds of household surveys, has already been described in detail.^17^ Briefly, we divided Lusaka district into 24 clinic catchment areas, each comprising a varying number of standard enumeration areas. For each survey round we selected three enumeration areas within each catchment area using probability-proportional-to-size sampling. Within each selected enumeration area, interviewers first located the approximate centre of the area. Then, working outward in a randomly selected direction, they selected households at regular intervals – such as every fifth household they encountered – until they had selected 50 households. If interviewers were unable to contact a respondent in a selected household after three attempts – or if consent for that household’s participation could not be obtained – they selected a neighbouring replacement household. In each of the 12 survey rounds, interviewers sampled 3600 households over a period of about three weeks.

Interviewers asked a member of each selected household to identify the household heads. If an adult male and an adult female were identified as joint heads of a selected household, the female head was selected for interview. This preferential selection of female heads of households was to facilitate comparison of our data with data collected in the Zambia Demographic and Health Surveys. The selected head was interviewed in English, Nyanja or Bemba. The interviewer described the objectives of the survey to the head and the head was asked for their written informed consent. Once consent had been given, the interviewer used a standardized questionnaire to collect detailed demographic and medical information about all members of the household – including the timing of any deaths that had occurred among household members within the previous 12 months. Interviewees were asked to state how many orphans under the age of 16 years were living in their households. They were also asked about their knowledge, attitudes and practices relating to HIV. As in the 2007 Zambia Demographic and Health Survey,^13^ interviewees were considered to have comprehensive knowledge of HIV if they correctly answered five questions about HIV transmission risk and so indicated that they knew that: HIV cannot be transmitted through mosquitoes, HIV cannot be transmitted by witchcraft, HIV transmission risk can be reduced through condom use, HIV transmission can be reduced by having one HIV-negative sex partner, and a healthy-looking person can have HIV.

Interviewees were asked if they had ever been tested for HIV and whether any non-pregnant members of their households were taking ART. The Institutional Review Board of the University of Zambia, the University of North Carolina at Chapel Hill and the University of Alabama at Birmingham approved the study protocol.

When planning our analysis, we assumed that most inhabitants of a specific catchment area would seek medical care at their local community clinic and that study households would only have access to ART when ART became available at that clinic. Our initial aim was therefore to compare trends in all-cause mortality at community level and to determine whether mortality trends remained the same before and after ART became available at the local clinic. However, a substantial proportion of interviewees in the first survey round reported that they had received medical care at clinics that were located outside their own communities.^17^ We therefore decided to focus on district-wide trends. As accurate cause-of-death information could not be collected from our interviewees, we studied all-cause mortality – rather than HIV-related mortality – as our primary outcome measure. To relate such mortality to the scale-up of the ART programme, we report the number of people actively enrolled in the ART programme in Lusaka district at the time of each survey round, as well as the cumulative enrolment.^18^^,^^19^

We tabulated the demographic characteristics of the study households and interviewees. For each survey round, we calculated rates of all-cause mortality as the numbers of deaths per 100 person–years – using the 12 months before the interview as the reference period. In these calculations, for each household member who had died in the 12 months before the interview and for each member who was reported to be less than 12 months of age at the time of the interview, we used the number of months that that member had been alive in the previous 12 months and 6 months as survival times, respectively.

To determine trends in mortality, we used a Poisson regression to estimate the mortality rate ratio – i.e. an estimate of the relative annual change in mortality – for the period 2004–2011. Next, we investigated this ratio for trend heterogeneity by sex and age group. The age groups we used – under 5, 5–14, 15–49 and over 50 years – were selected to facilitate comparison of our mortality data with those collected in Zambian Demographic and Health Surveys. We also calculated age-standardized mortality rates, using the INDEPTH Network’s population structure for sub-Saharan Africa for reference.^20^

In secondary analyses, we described trends in the respondents’ HIV-related knowledge, attitudes and practices. For each question on such knowledge, attitudes and practices, we calculated the proportion in each survey round of each possible response – i.e. yes, no or do not know. We then calculated the proportion of comprehensive HIV knowledge among the interviewees in each survey round. We also described trends in the proportions of households that included orphans and households with a non-pregnant member taking ART. We analysed prevalence trends for each question using separate generalized linear regression models that provided estimates of the mean yearly change between 2004 and 2011.

The values we report have been adjusted for the complex sampling design. We calculated standard errors and corresponding 95% confidence intervals (CI) using Taylor series linearization.^21^ Data were analysed using Stata 11.2 (StataCorp LP, College Station, United States of America).

## Results

Of the 43 200 households included in our analysis, 3800 (9%) were replacement households. Of the replacement households, 2650 (70%) were selected because a household head could not be contacted and 1088 (29%) because a household head declined to participate. Interviewees in the 43 200 households provided information on 207 798 household members. Table 1 contains details of the timing of the surveys, along with contextual information about the number of clinics providing ART and the number of patients receiving ART at the time of each survey.

**Table 1:** Survey schedule and antiretroviral therapy scale-up, Lusaka district, Zambia, 2004–2011

Survey round	Date	No. of public clinics dispensing ART^a^	% of district’s households in catchment areas of ART-dispensing clinics	No. of people enrolled in ART	
				Cumulative	At time of survey^b^
1	Nov 2004	9	47.9	5 478	5 017
2	Jun 2005	11	60.2	16 544	13 724
3	Nov 2005	11	60.2	24 256	18 739
4	Apr 2006	13	72.5	31 785	22 996
5	Mar 2007	16	82.7	50 921	31 967
6	Aug 2007	16	82.7	59 851	35 350
7	Nov 2007	16	82.7	65 669	38 012
8	Feb 2008	16	82.7	70 910	40 091
9	Sep 2010	19	88.0	131 572	63 358
10	Feb 2011	19	88.0	141 910	66 508
11	Jun 2011	19	88.0	149 342	67 693
12	Sep 2011	19	88.0	154 748	68 018

ART: antiretroviral therapy.

^a^ Throughout the study period there were 24 public clinics in Lusaka district.

^b^ Actively enrolled in the ART programme in Lusaka.

We only observed minor between-survey differences in the respondents’ sex, marital status and – for female interviewees – parity. Food security and employment increased over the study period whereas immigration and denial of medical care – because of the patients’ inability to pay for care – decreased (Table 2). The overall mean age of the respondents were 33.2 years (range between surveys: 32.2–34.3), they had attained education for 8.6 years (range between surveys: 8.0–9.5) and had an average of three living children (range between surveys: 2.9–3.2).

**Table 2:** Characteristics of household respondents, Lusaka district, Zambia, 2004–2011

Characteristic	Overall^a^	Minimum^a,b^	Maximum^a,b^
**Proportion of all respondents (*n* = 43 200) **			
Female	87.9	82.0 (11)	94.8 (7)
Married or living with partner	76.3	74.0 (11)	81.8 (7)
Giving birth in previous 12 months or partner of woman who had given birth in previous 12 months	13.6	11.3 (10)	16.2 (7)
Always or usually with enough food in the household	52.3	40.9 (1)	61.5 (10)
Denied medical care in previous 12 months because of inability to pay	8.8	4.4 (12)	15.0 (1)
In household that had moved to its present location in previous months	11.1	7.5 (7)	19.4 (1)
Attending local public clinic for health care	69.5	59.2 (11)	76.7 (7)
**Proportion of male respondents (*n* = 6059) with paid employment**	80.4	74.8 (2)	86.2 (10)
**Proportion of female respondents (*n* = 37 141) with paid employment**	40.0	32.2 (2)	45.0 (9)

^a^ The proportions are adjusted for the complex sampling design.

^b^ As recorded in any of the 12 survey rounds. The survey round in which each value was recorded is indicated in parentheses.

**Fig. 1:**
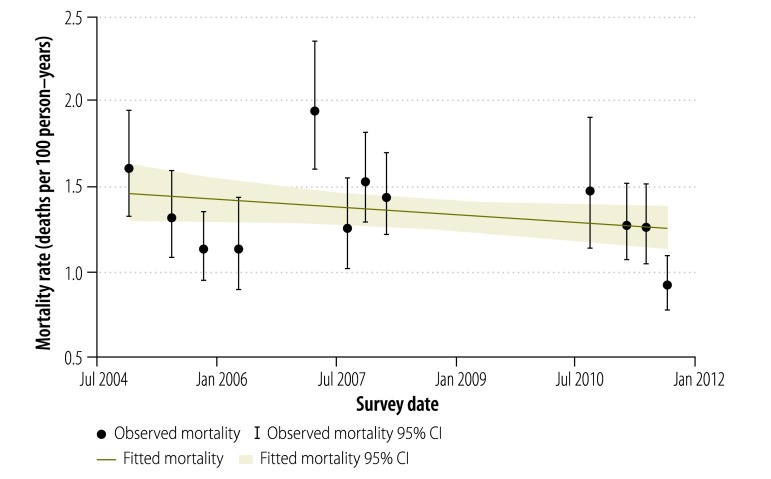
Observed and fitted all-cause mortality rates, Lusaka district, Zambia, 2004–2011

Across all survey rounds, household members contributed 204 263 person–years of survival and 2537 deaths. All-cause mortality was found to decrease modestly over time (mortality rate ratio: 0.98; 95% CI: 0.95–1.00; *P* = 0.093; Fig. 1) and a similarly small decrease was seen after the age-standardization of the data (mortality rate ratio: 0.98; 95% CI: 0.95–1.00; *P* = 0.083). Although there was clustering of mortality by enumeration area, the effective sample size for the analysis of mortality trend remained extremely large.

CI: confidence interval.

Note: The rates shown have not been age-standardized.

As there was no evidence of mortality trend heterogeneity by age group (*P* = 0.31) or sex (*P* = 0.50), we calculated stratum-specific mortality rates across the study period (Table 3). Household members between 15 and 49 years of age comprised 56% of the sample, and experienced a mean mortality of 1.33 (95% CI: 1.23–1.44) deaths per 100-person years. The corresponding mortality rate for the 15% of household members who were under 5 years of age was 2.14 (95% CI: 1.85–2.47) deaths per 100 person–years. Females comprised over half (53%) of the household members investigated. Female mortality was lower than male: 1.23 (95% CI: 1.14–1.33) versus 1.50 (95% CI: 1.39–1.63) deaths per 100 person–years.

**Table 3:** Mortality rates for household members by age group and sex, Lusaka district, Zambia, 2004–2011

Characteristic	Proportion of household members (%)	Mortality rate	
		Deaths/100 person–years	95% CI
**Age (years)**			
< 5	14.7	2.14	1.85–2.47
5–14	24.9	0.42	0.36–0.50
15–49	55.6	1.33	1.23–1.44
≥ 50	4.8	4.44	3.93–5.02
**Sex**			
Male	47.4	1.50	1.39–1.63
Female	52.6	1.23	1.14–1.33

CI: confidence interval.

**Fig. 2:**
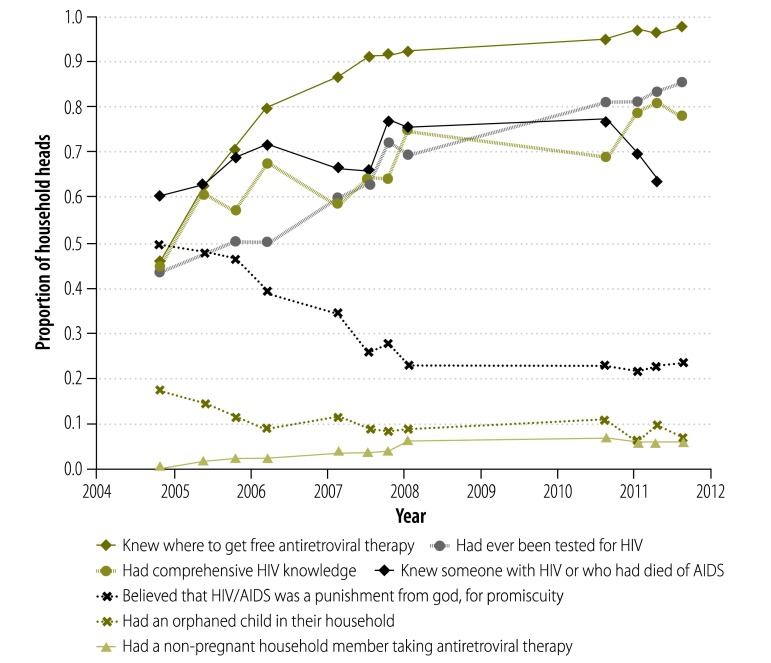
Human immunodeficiency virus-related knowledge and attitudes, as reported by household heads, Lusaka district, Zambia, 2004–2011

AIDS: acquired immunodeficiency syndrome; HIV: human immunodeficiency virus.

Over the first seven surveys, interviewees were increasingly likely to know where to get free ART. The proportion of interviewees who reportedly knew where to obtain such free therapy had approached 100% by the eighth survey and remained very high for the remainder of the study period. Across the whole study period, interviewees were increasingly likely to report that a non-pregnant household member was taking ART; to report that they had been tested for HIV; to have comprehensive HIV knowledge; or to know someone with HIV or someone who had died of AIDS. Conversely, the proportions of interviewees who reportedly believed that HIV/AIDS was “a punishment from God for promiscuity” and who reported the presence in their household of at least one orphan aged less than 16 years decreased during the study (Fig. 2; *P* < 0.001 for the trend in each measure).

## Discussion

Between 2004 and 2011, we documented positive trends in HIV-related knowledge, attitudes and practices across Lusaka district. These results align with the corresponding steady increase in individuals enrolled into HIV care and actively receiving ART. Although our study was timed to coincide with the roll-out of one of the largest urban ART programmes in Africa,^18^^,^^22^ these encouraging trends did not correlate with a substantial decline in all-cause mortality in our study area. We observed measurable increases in employment, food security, knowledge about HIV and health-care access and decreases in the proportion of households with orphan members. Taken together, these trends indicate an increase in the general well-being of the study population.

Our failure to observe a substantial reduction in mortality contrasts with the results of several studies conducted elsewhere in Africa. In rural South Africa, for example, researchers investigated the effects of an ART programme’s expansion over a 3-year period and observed reductions in mortality of more than 20% among adults and about 49% among young children.^6^^,^^7^ Similar reductions in adult mortality were observed in rural Malawi, in the 4 years after the expansion of an ART programme.^8^^,^^9^ In urban Ethiopia, Reniers et al. used data from burial site registries and reported reductions in adult mortality of more than 20% in the 3 years after ART had been introduced.^10^ Burial site surveillance was also used in Malawi, which showed a 37% reduction in all-cause mortality in the 5 years after ART scale-up.^11^ Finally, investigation of sibling survival data from Demographic and Health Surveys conducted in 27 African countries, including nine in which an HIV treatment programme funded by the United States President’s Emergency Plan for AIDS Relief was operating, found that the odds of death from any cause were 16% lower in these nine countries than in the 18 other countries.^12^

Our results also appear inconsistent with country-wide data from the World Bank and the Joint United Nations Programme on HIV and AIDS, which indicate that, in Zambia, there have been large declines in all-cause mortality since 2001^23^ and in HIV-related mortality since 2003.^14^

Mathematical models have demonstrated that low or delayed ART uptake will attenuate reductions in AIDS mortality^24^^–^^27^ and this may be applicable in Lusaka. However, there are several other possible reasons why we failed to observe a more substantial reduction in all-cause mortality. First, it is possible that increases in non-HIV-related mortality offset the expected declines in HIV-related mortality. We believe this to be unlikely, however, given the apparent improvements in interviewees’ socioeconomic status – e.g. in terms of employment and food security – and in their access to health care. Further, in Zambia the risk of death attributable to HIV is known to be extremely high.^28^

Second, our survey began shortly after a rapid scale-up began and without an opportunity to measure the mortality rate experienced immediately before the introduction of free ART services. The introduction of free ART may have rapidly led to substantial reductions in HIV-related mortality that we simply missed because they occurred before our first survey.

Third, the threshold of ART coverage necessary to produce major community-wide reductions in all-cause mortality may not have been reached yet. Early screening and entry into care are needed to ensure optimal survival for HIV-infected individuals. It was only early in 2010 that the Zambian ART programme adopted more inclusive eligibility criteria for ART, including a higher CD4+ lymphocyte threshold of 350 cells per mm^3^. In addition, greater programmatic focus is needed in the areas of patient adherence and retention. Attrition is high in the Lusaka ART programme,^18^^,^^29^ as in other ART programmes in sub-Saharan Africa.^30^^–^^32^ As mathematical models show, when many patients are lost to follow-up – and, therefore, discontinue therapy prematurely – the public health benefits of ART programme expansion are substantially reduced.

One encouraging result from our study was that the proportion of study households that held young orphans decreased from 17% in November 2004 to 7% in September 2011. This apparent decline in orphanhood is supported by the figures reported in Zambia’s Demographic and Health Surveys for 2001–2002 and 2007.^13^^,^^33^ Orphanhood has been shown to be associated with adult AIDS mortality.^34^^,^^35^

We also observed encouraging trends in HIV-related knowledge, attitudes and practices. This observation demonstrates the successful penetration of health communication messages about HIV among the residents of Lusaka. As comprehensive knowledge about HIV and of the location of ART services approaches universality among the adult residents of Lusaka, those residents are, presumably, increasingly prepared to access ART themselves – if needed – or to facilitate the entry of other residents into HIV care. The stigma associated with HIV appears to be on the wane in Lusaka. The proportion of interviewees who reported that a non-pregnant member of their household was receiving ART was only 0.8% in November 2004 but had risen more than 9-fold, to 7.4%, by September 2010.

The strengths of our study include the use of a standardized questionnaire, the large sample size and a study period that extended beyond the first few years after ART scale-up. Our study also had some important limitations. First, the study relied on data reported by self-identified heads of households and both the reporting and self-identification may be prone to bias. Any such bias is, however, likely to have been similar in each survey round and, reassuringly, the mean adult mortality that we recorded per 1000 person–years – 13.3 deaths – is similar to the corresponding values recorded in Zambia’s Demographic and Health Surveys for 2001–2002 and 2007: 14.1 and 12.5 deaths, respectively. Second, we made no attempt to investigate those members of the study households who died more than 12 months before the interviews or those who left study households – and, possibly, died – in the 12 months before the interviews. HIV-related immigration into a community and HIV-related emigration out of a community – as described in South Africa^36^ – can create unquantifiable bias in the estimation of mortality. Third, although HIV-related mortality would have been a more useful primary outcome in our study, we were unable to collect information regarding the cause or circumstances surrounding each death of interest. Fourth, although only 9% of the heads of households included in the initial selections of households for each survey round refused to participate in the study, such a level of non-participation could have been sufficient to alter our main findings – if the non-participating households had been systematically different from the participating households in terms of our primary and secondary outcomes. Finally, our sampling frame was based on the most recent and available census data for Lusaka, which were collected in 2000. These data do not necessarily reflect a population that has been highly dynamic and growing since the year 2000.

In conclusion, despite encouraging increases in comprehensive HIV knowledge and improved HIV-related attitudes and practices, the expansion of ART services in Lusaka between 2004 and 2011 coincided with only a modest reduction in all-cause mortality. While this finding was unexpected, it emphasizes several critical factors for improving the population-level impact of ART. Further expansion of coverage with ART services requires close monitoring and investment, particularly for patients who qualify for HIV treatment but have not yet become ill. Greater coverage needs to be accompanied by a more systematic application of effective measures to increase patient adherence and retention. Monitoring population-level outcomes – using resource-appropriate methods such as the one described here – should be implemented, to provide ongoing feedback on programme performance. Finally, investments in ART programmes – and, more broadly, in health-systems – must continue. In settings such as Lusaka, which have clearly benefited from successful education and outreach programmes, a realignment of the ART programme’s priorities may be needed to ensure maximal public health benefit.

## References

[1] van Sighem AI, Gras LA, Reiss P, Brinkman K, de Wolf F; ATHENA national observational cohort study. Life expectancy of recently diagnosed asymptomatic HIV-infected patients approaches that of uninfected individuals. AIDS. 2010;24(10):1527–35. 10.1097/QAD.0b013e32833a3946 20467289

[2] Zwahlen M, Harris R, May M, Hogg R, Costagliola D, de Wolf F, et al. Antiretroviral Therapy Cohort Collaboration. Mortality of HIV-infected patients starting potent antiretroviral therapy: comparison with the general population in nine industrialized countries. Int J Epidemiol. 2009;38(6):1624–33. 10.1093/ije/dyp306 19820106 PMC3119390

[3] Antiretroviral therapy for HIV infection in adults and adolescents: recommendations for a public health approach. 2010 revision. Geneva: World Health Organization; 2010. Available from: http://whqlibdoc.who.int/publications/2010/9789241599764_eng.pdf[cited 2014 May 27]. 23741771

[4] Blacker J. The impact of AIDS on adult mortality: evidence from national and regional statistics. AIDS. 2004;18 Suppl 2:S19–26. 10.1097/00002030-200406002-00003 15319740

[5] Timaeus IM, Jasseh M. Adult mortality in sub-Saharan Africa: evidence from Demographic and Health Surveys. Demography. 2004;41(4):757–72. 10.1353/dem.2004.0037 15622953

[6] Herbst AJ, Cooke GS, Bärnighausen T, KanyKany A, Tanser F, Newell ML. Adult mortality and antiretroviral treatment roll-out in rural KwaZulu-Natal, South Africa. Bull World Health Organ. 2009;87(10):754–62. 10.2471/BLT.08.058982 19876542 PMC2755311

[7] Ndirangu J, Newell ML, Tanser F, Herbst AJ, Bland R. Decline in early life mortality in a high HIV prevalence rural area of South Africa: evidence of HIV prevention or treatment impact? AIDS. 2010 20;24(4):593–602. 10.1097/QAD.0b013e328335cff5 20071975 PMC4239477

[8] Jahn A, Floyd S, Crampin AC, Mwaungulu F, Mvula H, Munthali F, et al. Population-level effect of HIV on adult mortality and early evidence of reversal after introduction of antiretroviral therapy in Malawi. Lancet. 2008;371(9624):1603–11. 10.1016/S0140-6736(08)60693-5 18468544 PMC2387197

[9] Floyd S, Molesworth A, Dube A, Banda E, Jahn A, Mwafulirwa C, et al. Population-level reduction in adult mortality after extension of free anti-retroviral therapy provision into rural areas in northern Malawi. PLoS ONE. 2010;5(10):e13499. 10.1371/journal.pone.0013499 20976068 PMC2957442

[10] Reniers G, Araya T, Davey G, Nagelkerke N, Berhane Y, Coutinho R, et al. Steep declines in population-level AIDS mortality following the introduction of antiretroviral therapy in Addis Ababa, Ethiopia. AIDS. 2009;23(4):511–8. 10.1097/QAD.0b013e32832403d0 19169138 PMC2666986

[11] Mwagomba B, Zachariah R, Massaquoi M, Misindi D, Manzi M, Mandere BC, et al. Mortality reduction associated with HIV/AIDS care and antiretroviral treatment in rural Malawi: evidence from registers, coffin sales and funerals. PLoS ONE. 2010;5(5):e10452. 10.1371/journal.pone.0010452 20454611 PMC2864258

[12] Bendavid E, Holmes CB, Bhattacharya J, Miller G. HIV development assistance and adult mortality in Africa. JAMA. 2012;307(19):2060–7. 10.1001/jama.2012.2001 22665105 PMC3434229

[13] Zambia: demographic and health survey, 2007. Calverton: Macro International Inc.; 2009.

[14] Epidemiological factsheet: Zambia [Internet]. Geneva: Joint United Nations Programme on HIV and AIDS; 2011. Available from: http://www.unaids.org/en/Regionscountries/Countries/Zambia/[cited 2011 Jul 30].

[15] AIDSinfo [Internet]. Geneva: Joint United Nations Programme on HIV and AIDS; 2013. Available from: http://www.unaids.org/en/dataanalysis/datatools/aidsinfo/[cited 2013 May 22].

[16] 2010 census of population and housing. Lusaka: Republic of Zambia Central Statistical Office; 2011. Available from: http://unstats.un.org/unsd/demographic/sources/census/2010_phc/Zambia/PreliminaryReport.pdf[cited 2014 May 27].

[17] Giganti MJ, Levy JW, Banda Y, Kusanthan T, Sinkala M, Stringer JS, et al. Methods and baseline results of a repeated cross-sectional survey to assess the public health impact of antiretroviral therapy in Lusaka, Zambia. Am J Trop Med Hyg. 2010;82(5):971–7. 10.4269/ajtmh.2010.09-0739 20439984 PMC2861382

[18] Stringer JS, Zulu I, Levy J, Stringer EM, Mwango A, Chi BH, et al. Rapid scale-up of antiretroviral therapy at primary care sites in Zambia: feasibility and early outcomes. JAMA. 2006;296(7):782–93. 10.1001/jama.296.7.782 16905784

[19] Fusco H, Huschman T, Mbweeta V, Chi B, Levy J, Sinkala M, et al. Electronic patient tracking supports rapid expansion of HIV care and treatment in resource-constrained settings [Internet]. Geneva: International AIDS Society; 2005. Available from: http://www.iasociety.org/Default.aspx?pageId=11&abstractId=2177493[cited 2014 June 1].

[20] INDEPTH Network. Population, health and survival at INDEPTH sites. Population and health in developing countries. Volume 1 Ottawa: International Development Research Centre; 2002.

[21] Heeringa S, West BT, Berglund PA. Applied survey data analysis. London: Chapman and Hall; 2010 10.1201/9781420080674

[22] Bolton-Moore C, Mubiana-Mbewe M, Cantrell RA, Chintu N, Stringer EM, Chi BH, et al. Clinical outcomes and CD4 cell response in children receiving antiretroviral therapy at primary health care facilities in Zambia. JAMA. 2007;298(16):1888–99. 10.1001/jama.298.16.1888 17954540

[23] World Databank [Internet]. Washington: World Bank; 2011. Available from: http://databank.worldbank.org/ddp/home.do[cited 2011 Jul 30].

[24] Abbas UL, Anderson RM, Mellors JW. Potential impact of antiretroviral therapy on HIV-1 transmission and AIDS mortality in resource-limited settings. J Acquir Immune Defic Syndr. 2006;41(5):632–41. 10.1097/01.qai.0000194234.31078.bf 16652038

[25] Granich RM, Gilks CF, Dye C, De Cock KM, Williams BG. Universal voluntary HIV testing with immediate antiretroviral therapy as a strategy for elimination of HIV transmission: a mathematical model. Lancet. 2009;373(9657):48–57. 10.1016/S0140-6736(08)61697-9 19038438

[26] Grangeiro A, Escuder MM, Menezes PR, Alencar R, Ayres de Castilho E. Late entry into HIV care: estimated impact on AIDS mortality rates in Brazil, 2003–2006. PLoS ONE. 2011;6(1):e14585. 10.1371/journal.pone.0014585 21283618 PMC3026775

[27] Walensky RP, Wood R, Weinstein MC, Martinson NA, Losina E, Fofana MO, et al.; CEPAC-International Investigators. Scaling up antiretroviral therapy in South Africa: the impact of speed on survival. J Infect Dis. 2008;197(9):1324–32. 10.1086/587184 18422445 PMC2423492

[28] Dzekedzeke K, Siziya S, Fylkesnes K. The impact of HIV infection on adult mortality in some communities in Zambia: a cohort study. Trop Med Int Health. 2008;13(2):152–61. 10.1111/j.1365-3156.2007.01985.x 18304260

[29] Chi BH, Cantrell RA, Mwango A, Westfall AO, Mutale W, Limbada M, et al. An empirical approach to defining loss to follow-up among patients enrolled in antiretroviral treatment programs. Am J Epidemiol. 2010;171(8):924–31. 10.1093/aje/kwq008 20219765 PMC2850972

[30] Rosen S, Fox MP, Gill CJ. Patient retention in antiretroviral therapy programs in sub-Saharan Africa: a systematic review. PLoS Med. 2007;4(10):e298. 10.1371/journal.pmed.0040298 17941716 PMC2020494

[31] Fox MP, Rosen S. Patient retention in antiretroviral therapy programs up to three years on treatment in sub-Saharan Africa, 2007–2009: systematic review. Trop Med Int Health. 2010;15 Suppl 1:1–15. 10.1111/j.1365-3156.2010.02508.x 20586956 PMC2948795

[32] Rosen S, Fox MP. Retention in HIV care between testing and treatment in sub-Saharan Africa: a systematic review. PLoS Med. 2011;8(7):e1001056. 10.1371/journal.pmed.1001056 21811403 PMC3139665

[33] Zambia demographic and health survey 2001–2002. Calverton: Macro International Inc.; 2003.

[34] Watts H, Lopman B, Nyamukapa C, Gregson S. Rising incidence and prevalence of orphanhood in Manicaland, Zimbabwe, 1998 to 2003. AIDS. 2005;19(7):717–25. 10.1097/01.aids.0000166095.62187.df 15821398

[35] Grassly NC, Timaeus IM. Methods to estimate the number of orphans as a result of AIDS and other causes in Sub-Saharan Africa. J Acquir Immune Defic Syndr. 2005;39(3):365–75. 10.1097/01.qai.0000156393.80809.fd 15980700

[36] Welaga P, Hosegood V, Weiner R, Hill C, Herbst K, Newell ML. Coming home to die? The association between migration and mortality in rural South Africa. BMC Public Health. 2009;9(1):193. 10.1186/1471-2458-9-193 19538717 PMC2706824

